# Communications

**Published:** 1902-03

**Authors:** 


### COMMUNICATIONS.

### DISCUSSION OF "COLUMELLA'S" PROPOSITION FOR THE CONTROL OF TUBERCULOSIS.

EDITOR OF THE JOURNAL OF COMPARATIVE MEDICINE, ETC.

SIR: In answer to your personal request for an opinion on "Columella's" proposition, I would say that it is less a matter demanding opinions than one to be submitted to the test of practical experiment. From "Columella's" stand-point it is altogether desirable and could be made a practical success. The fundamental principles involved have already been successfully applied not in Denmark alone, but also in the ranges of the Northwest and in a number of private herds.

But I would submit that "Columella's" view of his particular side of the pillar does not give us an adequate view of the pillar as seen from all sides in succession and as a whole. The conditions met with on some of the other sides may possibly play havoc with the system of "Columella" when the attempt is made to put it in practice. The proposition has to deal not only with the problem of utilizing slightly tuberculous cows and the raising of healthy progeny from them, but quite as much with the disposition of competent and trustworthy men to take up tuberculous dairying as a business and that of the general public toward the consumption of dairy products which are certified to be from tuberculous cows. Some of the necessary attendant conditions which would involve drawbacks to the practical working of the plan might be placed under successive headings:

1. The difficulty of finding the right kind of men to take hold of the dairies of tuberculous animals as a personal investment. Men of good judgment are necessary, but this class is not likely to seek the investment. The tuberculous cows are less thrifty than sound cows, and require better feeding and care to keep them in a condition and up to a milk yield approximating that of the sound herds. Other things being equal, there will be more losses in a tuberculous herd than in a sound one; then as the milk rests under a cloud its value in the market must be considerably depreciated. There is little to encourage a dairyman to enter on such a speculation.

In the Northwest, where the tuberculous animals are used to raise progeny only, where the cost of maintenance is almost *nil*, where the stock live in the open air, and where the matured offspring are sold on their own individual evidence of prime condition and sound health, the case is essentially different. Again, in Denmark, with its general prevalence of tuberculosis in bovine herds, and with the habit of keeping the tuberculous cows on the same farm as the others, only in separate inclosures, the public apprehension is lessened, and there is a ready market for the milk. The experience of Pennsylvania, however, seems to show that in American communities the same measure of indifference cannot be counted on as in Denmark.

2. The concentration in one herd of all the tuberculous cows will, *cæteris paribus*, serve to increase the infection in the building, lessen and depreciate the milk product, and multiply the losses of cattle. True, special precautions may be taken to counteract some of these evil results, but all such enhance the expense of conducting the establishment and lessen the profits or increase the losses of the owner.

3. The office outlay of the government in tracing and recording all sales and other transactions in the tuberculous cattle, and the cost of frequent veterinary supervision and testing, of licensing, branding, etc., will be no inconsiderable item to be added to an enterprise which is already seriously handicapped.

4. The sterilized milk cannot be justly or honorably sold as normal milk. It has the inevitable drawback which attaches to all sterilized or even Pasteurized milk—the constant suspicion of the presence of dead bacilli and the certain presence of minute quantities of tuberculin, which, however small in amount, must be the greater that every cow in the herd has tuberculosis and is producing the toxic matters. To certify this as good milk is a wrong to the innocent consumer which no government surely would be hardy enough and reckless enough to do, lest it should be swept out of existence in a storm of indignation.

To certify the sterilization would be to assign the milk to a lower and cheaper grade, while to fail to certify at all would be to throw suspicion on the whole milk product of the State in which tuberculous dairies are licensed.

These are some of the things that appear to me on the other sides of "Columella's" pillar, and which must fully be considered in entering on his project. Under given conditions his proposal would be a feasible one. In our Eastern States it may be questioned whether it would be profitable.

JAMES LAW.

### CONNECTICUT CRITICISES "COLUMELLA."

EDITOR JOURNAL OF COMPARATIVE MEDICINE, ETC.:

I NOTE in the January issue of the JOURNAL an article entitled "A Proposal of a New Plan for Controlling Tuberculosis of Cattle Conservatively." Moreover, I found an editorial soliciting a discussion of the same plan. My interest was awakened owing to my earlier connection with this work and a knowledge of the entire lack of adequate means in this State of preventing the spread of tuberculosis.

The State has, indeed, an individual appointed by the Governor as a Cattle Commissioner, and, as far as the power he is invested with permits him, he is endeavoring to control the disease.

The plan proposed by "Columella" as drafted and explained is worthy of considerable praise, and shows a desire to accomplish the greatest good to the greatest number. It appears to me, however, in the face of many obstacles, practically impossible of adoption, especially as a measure of extensive benefit. Experience in connection with this disease leads me to believe there are a large number of cattle owners who claim not to believe in tuberculin. It is a question whether this claim is based entirely upon the actual fear of the physical injury to live-stock; indeed, I do not believe that this opposing force actually thinks this. That such advocates have considerable weight is developed by the opposition of the dealers, dairymen, and, to some extent, the breeders in this State, whose influence was so great that, in the face of extensive object lessons and no compulsion to an examination of the herds (the work having been done entirely upon solicitation), laws were enacted forbidding any animal to be condemned as tuberculous, for State remuneration, if so condemned by the use of tuberculin as a diagnostic agent. Of course, it may be said that time will remedy this evil. Possibly. I will endeavor to show why it will be difficult. There are several reasons why those connected with breeding, bartering, and dairying wish to prevent tuberculin adopted as a means of diagnosis, and from reasons given out from these several sources will come the impossibilities.

Owners of pure-bred registered stock have learned that by the use of tuberculin valuable animals in their herds react. It is, for them at least, difficult to believe disease exists, so healthy do these suspected animals appear. Now these animals are valuable as producers of a strain of milkers for sale and registration ; breeders are loath to part with such animals. Many are held at fancy prices and realizing a comfortable profit; the licensed dealer would be utterly unable to offer sufficient inducements. Moreover, should the breeder divide his business in raising healthy and tuberculous cattle, he at once brands his herd as diseased and causes to himself a considerable financial loss. Again, were he to sacrifice these animals to a dealer, too much would depend upon the integrity of the latter, and then would develop a source of contagion influenced by the thirst for gain.

The ordinary cattle dealer, the principal party that "Columella" proposes to entrust with a license, must become a conscientious dealer. He must be induced to offer enough money for his purchases to influence the dairyman, as well as the breeder, to dispose of his fine milkers. The dairyman has a large milker that has been tested and is shown to be tuberculous; but she appears healthy; he realizes that had no test been performed everything would have remained quiet and a profit was to be realized from her products. Can the licensed dealer pay enough to enable the dairyman to replace her without loss? He must pay enough for the farmer to overcome the chance of losing money after testing the herd. Unless the dealer does this the dairyman must continue to keep a possible source of infection to healthy stock until she has commenced to go dry.

The dealer, it seems to me, cannot offer the dairyman the true producing value, for it is doubtful if he can find a ready market for her or her products, and this brings me to the last great reason of the objection to "Columella's" plan, viz.: "The value of the marketable products of tuberculous cattle as compared with healthy." And the ordinary herds to-day have health certificates, from a producer's stand-point at least, founded on the saying "ignorance is bliss."

Producers of milk from diseased cattle would find a slow market. Creameries could not afford to advertise that their cream is gathered from diseased herds, even though they insist and explain that it has been rendered safe for use, for, competition stimulating trade, someone will at once advertise milk from healthy stock, direct from the cow, and harmless without the sterilization required, and, though "Columella" states that there are no valid grounds for not accepting his scheme, public opinion will furnish a difficult problem.

The consumer must be taught that milk from diseased animals treated is as nice and as valuable as pure milk. Moreover, the extra handling will demand a better price or the producer must be contented with a smaller profit.

Will not the producer handling milk from healthy stock endeavor to protect himself? Will he not have a right to demand laws like those in reference to oleomargarine? He certainly will do so if progressive, and milk of diseased animals will have to be acknowledged. Here again must the public depend too much on the holder of the license. All this cheapens a more expensive article.

The sale of beef is another problem. Under the present laws beef can be obtained with a government guarantee of freedom from disease, this cheapens the market for native diseased flesh and will add another burden to the poor.

Should the body be too greatly diseased the dealer loses a larger proportion of his investment, or will be encouraged to purchase such stock to deliver or sell to the State at a small profit, ridding the State of bad cases, to be sure, but all the while encouraging the spread of disease, dishonesty, and excessive expense to the municipal authorities without systematic work.

RICHARD P. LYMAN, M.D.V.

